# Supplementary figures and images for: Mobile Introns Shape the Genetic Diversity of Their Host Genes
Source: Genetics. 2017 Feb 13;205(4):1641–8. doi: 10.1534/genetics.116.199059 (PMC5378118; doi:10.1534/genetics.116.199059)

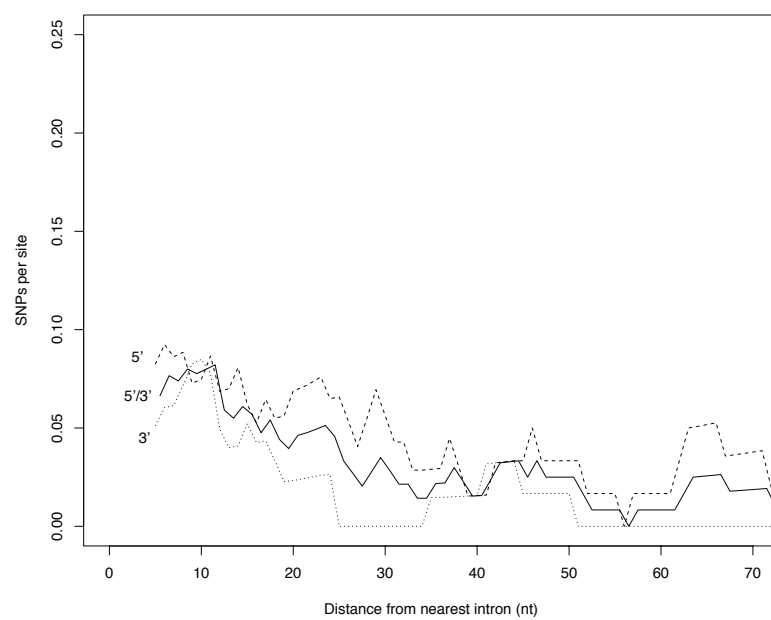

Figure S 1 – Elevated SNP density at mitochondrial 5' and 3' intron-exon boundaries in *S. cerevisiae*.

Supplement: Supplementary file 1 [file 1641file001.pdf]
